# Pathogens on fire: a scoping review of smoke-borne pathogen ecology in the One Health framework

**DOI:** 10.7717/peerj.20605

**Published:** 2026-01-22

**Authors:** Ashish Adhikari, Nattapol Kraisitudomsook, Krista L. Bonfantine, Phinehas Lampman, Sam Fox, Jason A. Smith, Borna Mehrad, Leda N. Kobziar, Karen A. Garrett

**Affiliations:** 1 Emerging Pathogens Institute, University of Florida, Gainesville, FL, United States of America; 2 Global Food Systems Institute, University of Florida, Gainesville, FL, United States of America; 3 Department of Plant Pathology, Institute of Food and Agricultural Sciences, University of Florida, Gainesville, United States of America; 4 Department of Microbiology, Faculty of Science, Mahidol University, Bangkok, Thailand; 5 Department of Forests, Rangeland, and Fire Sciences, University of Idaho, Coeur d’Alene, ID, United States of America; 6 Department of Biological and Environmental Sciences, University of Mount Union, Alliance, OH, United States of America; 7 Department of Medicine, University of Florida, Gainesville, FL, United States of America

**Keywords:** Wildland fires, Wildfires, Smoke-borne pathogens, Disease ecology, Microbiomes, Pathogens, Microbes, Dispersal, One health, Wind dispersal

## Abstract

**Background:**

Wildland fires are increasing in both frequency and severity in many areas globally. Smoke from wildland fires (wildfires and prescribed burns), as well as agricultural burning, releases not only pollutants but also viable microorganisms, including pathogens capable of long-distance dispersal, potentially posing unrecognized risks to human, animal, and plant health.

**Objectives:**

This scoping review synthesizes knowledge about pathogenic microbial dispersal in smoke from wildland fires, identifies gaps in pathogen ecology and epidemiology, and outlines research priorities in a One Health framework.

**Methods:**

This review followed the Arksey & O’Malley framework with PRISMA-ScR guidance, using systematic searches in PubMed, Google Scholar, and grey literature sources (USDA Forest Service, World Health Organization, U.S. Environmental Protection Agency). After screening and applying inclusion criteria, 36 studies were retained that addressed microbial transport, viability, and disease associated with wildland fire smoke.

**Results:**

There is evidence that wildland fire smoke can aerosolize diverse microbial assemblages, including pathogenic fungi such as *Coccidioides* and *Puccinia*, and bacteria capable of forming heat-resistant spores. If microbes can remain viable in smoke across greater distances, there would be the possibility of long-distance dispersal while suspended in smoke plumes. However, data about infection outcomes, dose–response relationships, and host susceptibility are lacking. Current wildland fire smoke surveillance focuses almost exclusively on abiotic pollutants, leaving microbial risks largely ignored.

**Conclusions:**

A One Health approach integrates fire ecology, aerobiology, microbiology, and epidemiology across host species. After determining how important the role of dispersal in smoke is for human, animal, and plant health, priority actions may include improving pathogen viability sampling, incorporating microbial monitoring into smoke surveillance networks, and developing predictive models to assess health and ecological risks.

## Introduction

### Smoke-borne pathogens in the One Health framework

In many regions around the world, wildland fires are increasing in frequency, size, or severity due to climate change, altered land management, fuel accumulation, and changes in ignition patterns (Abatzoglou et al., 2021; Higuera & Abatzoglou, 2021; Pausas & Keeley, 2021; Westerling et al., 2006). For example, climate-fire models project a doubling of area burned in western US forests compared to 1991–2020 in the near-term (2021–2050), assuming constant fuel levels (Abatzoglou et al., 2021). A sharp increase in wildland fires in North America between 1960 and 2000 accelerated the release of airborne pollutants (Page et al., 2002), with Childs et al. (2022) reporting that the number of people in the US exposed to >1 day of harmful levels of wildland fire smoke over the last decade has increased 27-fold. Wildland fires emit massive quantities of particulate matter, gases, and potentially substantial quantities of microbes (*e.g.*, bacteria and fungi (Mims & Mims, 2004)), altering atmospheric composition and potentially posing risks to human, animal, and plant health (Etherton et al., 2024; Hauser et al., 2021; Kobziar & Thompson III, 2020; Kobziar et al., 2022; Kobziar et al., 2024a). Wildfire-specific PM_2.5_ was associated with higher respiratory hospital admissions than non-wildfire PM_2.5_, with a 1.3–10% rise per 10 µg/m^3^ increase compared to 0.67–1.3% for other sources (Aguilera et al., 2021). While wildland fire smoke has been extensively studied in terms of air pollutants (*e.g.*, particulate matter (PM), ozone, toxic gases), there is growing evidence that viable microorganisms, including bacterial and fungal pathogens, can be aerosolized (Kobziar et al., 2018; Kobziar et al., 2022; Kobziar et al., 2024b; Bonfantine et al., 2024; Mims & Mims, 2004) and transported over long distances (Yue et al., 2025). However, the ecology and epidemiology of smoke-borne microbes remain poorly understood, raising important questions about smoke’s role in disease emergence, pathogen dispersal, and environmental feedback mechanisms.

The One Health paradigm integrates threats to all types of hosts, including humans, domestic and wild animals, and plants. It offers “a collaborative, multisectoral, and transdisciplinary approach with the goal of achieving optimal health outcomes recognizing the interconnection between people, animals, plants, and their shared environment” (Mackenzie & Jeggo, 2019). The One Health definition, developed by the One Health High-Level Expert Panel (OHHLEP), states that One Health is an integrated, unifying approach that aims to sustainably balance and optimize the health of people, animals and ecosystems. It recognizes that the health of humans, domestic and wild animals, plants, and the wider environment (including ecosystems) are closely linked and inter-dependent (World Health Organization, 2021). Wildland fire smoke can carry living microbes that may infect humans, animals, and plants, even far from fire zones, making a One Health perspective important for tracking and managing these cross-domain threats (Kobziar & Thompson III, 2020).

Wildfire smoke can carry viable fungal and bacterial pathogens, and these microbes can travel long distances in smoke plumes, posing health risks to humans, animals, and plants across ecological and geographic boundaries (Kobziar et al., 2018; Kobziar & Thompson III, 2020). While direct yield impacts from smoke-borne microbes remain unproven, the hypothesis is grounded in the substantial burden of plant pathogens on global agriculture (Savary et al., 2019) and established biological effects of smoke on vertebrate health (Sanderfoot et al., 2022). Although no study directly evaluated *viruses* in wildfire smoke, exposure to wildfire particulate matter is associated with increased cases or deaths from COVID-19 outcomes (Navarro et al., 2021; Mahendran et al., 2025; McHale et al., 2025). In human health, Phillips et al. (2023) raised concern that large fires in regions with ongoing local transmission of the soil fungus *Coccidioides* spp. may be associated with increases in Valley Fever (coccidioidomycosis) incidence. Mulliken et al. (2023) reported a 20% increase in hospital admissions for coccidioidomycosis in the month following wildland fire smoke exposure in California. A meta-analysis reported that each 10 µg/m^3^ increase in short-term wildfire-related PM_2.5_ led to a 15% rise in COVID-19 infections, a 3% increase in respiratory infections, and a 20% increase in hospitalizations for systemic fungal diseases like coccidioidomycosis (Mahendran et al., 2025), highlighting the interconnected health risks from wildfires. Smoke from wildland fires carries human pathogens and allergens (Kobziar et al., 2018; Kobziar et al., 2022), with the potential to cause compounded health risks in humans (Hauser et al., 2021) and livestock (Sanderfoot et al., 2022) when combined with abiotic stressors in smoke.

Prescribed burning is a common practice in agricultural systems such as sugarcane production, and rRNA sequencing of fungal genes has shown that plant pathogenic genera such as *Puccinia*, *Ustilago, Aspergillus*, and *Candida* are aerosolized during prescribed fires (Kobziar et al., 2022). Bacterial pathogens documented in wildland fire smoke include, for example, *Bacillus anthracis-cereus, Pseudomonas syringae, Streptococcus* spp., *Escherichia-Shigella coli, Corynebacterium jeikeium, Acinetobacter ursingii,* and *Haemophilus haemolyticus influenzae* (Kobziar et al., 2022). Both fuel type and combustion phase (*i.e.,* smoldering *versus* flaming combustion) can modify emissions and associated health risks. For example, Kim et al. (2018) reported that smoke from flaming burns of peat, eucalyptus, pine, oak, and pine needles was more toxic and mutagenic per unit of particulate matter than smoke from smoldering burns, especially for peat and eucalyptus; however, when adjusted for the amount of smoke released, smoldering pine needles were the most mutagenic and smoldering eucalyptus was the most toxic. Although many studies have examined the transport of airborne human/animal pathogens in wind and dust storms (*e.g.*, Griffin, 2007; Triadó-Margarit, Cáliz & Casamayor, 2022), dispersal of fungal and bacterial pathogens in wildland fire smoke is an emerging research area (Kobziar & Thompson III, 2020). The complex combination of microbial dispersal that alters microbiomes, and the potential for increased risks of disease due to wildland fire smoke, motivates collaboration in a One Health framework among fire ecologists, microbiologists, epidemiologists, and public health experts.

**Figure 1:**
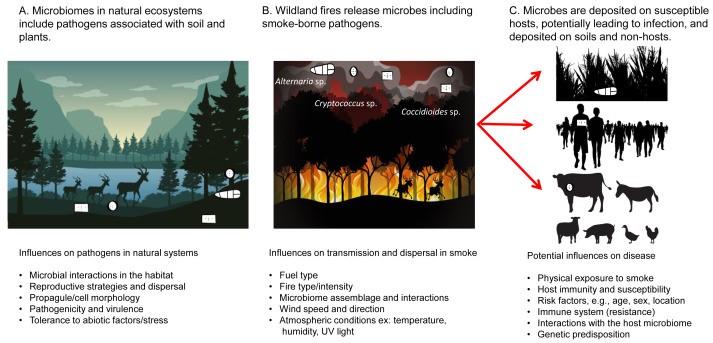
Smoke-borne pathogen ecology addresses the release of pathogens from soil and plant material in wildland fires, pathogen dispersal in smoke, deposition, and potential infection of a range of hosts. The One Health paradigm addresses pathogen communities important across hosts.

Pyroaerobiology—the study of the aerosolization and transport of viable microorganisms *via* wildland fire smoke—integrates microbiology and aerobiology, smoke and atmospheric sciences, fire behavior, and fire ecology (Kobziar et al., 2018). Smoke plumes can lift and disperse diverse microbial assemblages (Kobziar et al., 2018; Kobziar et al., 2024b), including spore-forming fungi and heat-resistant bacteria. The microbial assemblages detected in smoke indicate the potential for dispersal of microbes affecting a wide range of hosts and their communities, as laboratory experiments have shown that bacteria dispersed from fuels in smoke can colonize smoke-inundated soils (Ellington et al., 2024). Initial studies in pyroaerobiology reported that wildland fire smoke can contain up to four times more viable microbial cells than background air, with higher diversity metrics in both bacterial and fungal smoke communities (Kobziar et al., 2018; Kobziar et al., 2022; Moore et al., 2020; Bonfantine et al., 2024; Ellington et al., 2024). These findings highlight important One Health concerns, and the need for a review that integrates fire ecology, microbiology, and public health to map how microbes survive combustion, aerosolize, and disperse *via* smoke. Here, we define smoke-borne pathogen ecology as the study of pathogens in microbial assemblages and their interactions in fuel (soils and plants) and in the smoke from wildland fires, including their potential to infect hosts including humans, animals, and plants (Fig. 1). The objectives of this scoping review are to (1) synthesize existing research on smoke-borne pathogens, (2) identify the current knowledge gaps in pathogen ecology (including dispersal and other aspects of epidemiology) related to wildland fires, and (3) propose future research directions in a One Health framework. This scoping study of smoke-borne pathogen ecology across environmental, and public health domains supports the development of comprehensive strategies to understand and monitor potential infectious disease impacts from wildland fires.

### Methods

This scoping review follows the framework of Arksey & O’Malley (2005) and integrates elements from PRISMA-ScR (Tricco et al., 2018) to map existing knowledge about smoke-borne pathogen ecology (with a PRISMA scoping review checklist in Table S1). Building on the development of pyroaerobiology (Kobziar et al., 2018), this review explores microbial dispersal *via* wildland fire smoke and potential One Health implications for humans, animals, and plants.

**Figure 2:**
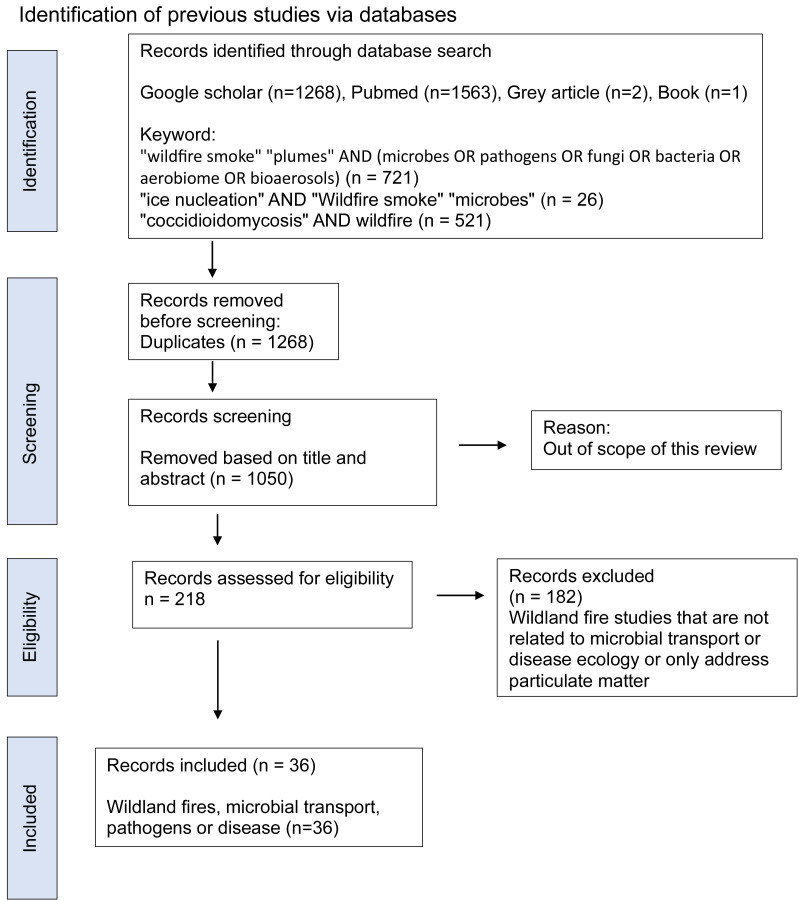
PRISMA flow diagram of the scoping review process based on the PRISMA-ScR extension, showing the literature search across Google Scholar, PubMed and grey literature sources (USDA FS, WHO, EPA), duplicate removal, title/abstract and full-text screening.

A targeted literature search was conducted across Google Scholar and PubMed, alongside grey literature sources such as technical reports from the USDA Forest Service, World Health Organization (WHO), and the US Environmental Protection Agency (EPA). Core keyword blocks included (1) “wildfire smoke” OR “smoke plumes” AND (microbes OR pathogens OR fungi OR bacteria OR aerobiome OR bioaerosols), (2) “ice nucleation” AND “wildfire smoke” “microbes”, and (3) “coccidioidomycosis” AND wildfire (PRISMA flow diagram, Fig. 2). No limit on the range of publication dates was applied in the literature search, but relevant studies were primarily within the last two decades. We did not find relevant articles in languages other than English, though we did not specifically exclude other languages. The most recent search was implemented on March 15, 2025.

The PRISMA methodology was employed to filter and select the relevant studies. We included studies that investigated pathogen transport in smoke from wildland fires (*e.g.*, prescribed fires, wildfires) or burning of agricultural fields, or provided empirical or conceptual insights into microbial presence and viability in smoke or smoke-polluted air, or explored atmospheric and ecological factors affecting pathogen dispersal or disease ecology related to smoke. Studies focused solely on-air pollution (*e.g.*, PM) without biological data, or lacking in relevance to pathogen or disease ecology, were excluded. We processed resulting articles from all databases and screened titles, abstracts and full text against the inclusion criteria. Disagreements on inclusion or exclusion were resolved through discussion among authors; no third parties were employed. We eliminated duplicate studies and those that did not meet the selection criteria or were outside the scope of this review. The 36 final studies obtained were focused on wildland fires (fire behavior, emissions and interactions, microbial transport, and disease association).

We summarize the research based on pathogen type (fungi, bacteria, viruses), fire characteristics (fire type and behavior if known, source fuel type or ecosystem, geographic location), and health/ecological implications. We used a narrative synthesis approach using descriptive and textual explanation to categorize findings into key themes: pathogen dispersal mechanisms, microbial viability, health risks, and future surveillance strategies. Since the objective was to map research gaps rather than critically evaluate study quality, no formal risk-of-bias assessment was conducted (Levac, Colquhoun & O’Brien, 2010). None of the studies in the scoping study reported a conflict of interest. By identifying gaps in scientific understanding of smoke-borne pathogen ecology, this review expands on the pyroaerobiology framework (Kobziar et al., 2018; Kobziar et al., 2022; Kobziar et al., 2024a; Kobziar et al., 2024b; Bonfantine et al., 2024; Ellington et al., 2024) and provides a foundation for future predictive modeling, risk assessment, and monitoring strategies for potential pathogen transport driven by the ∼6,440 Tg of biomass burned in wildland fires globally each year (Andreae, 2019).

We combined systematic database searches with grey literature sources and a dual-reviewer screening process, with the aim of improving understanding of pyroaerobiology by bringing together data from across multiple disciplines. Our goal is to provide a clearer view of how wildfire smoke may transport viable pathogens and the potential implications for One Health (Table 1), strengthening the evidence base for public health preparedness and ecological risk assessment.

**Table 1:** References reporting key results related to smoke-borne pathogens.

**Reference**	**Study System/Type of Fire**	**Microbial Groups/ Especially Pathogens**	**Main Results**
Parmeter Jr & Uhrenholdt (1976)	Pine litter or grass smoke exposure (lab chamber study)	Fungi	Substrates exposed to smoke for 1–16 min had inhibited germination of spores for several fungi and mycelial growth was arrested
Mims & Mims (2004)	Biomass fires (grass/leaves/twigs in backyard piles), Texas, USA	Bacteria and fungi	Viable fungal spores (including genera *Alternaria*, *Cladosporium*, *Fusariella*, *Curvularia*) and bacteria detected in smoke from biomass fires
Rajput, Anjum & Gupta (2017)	One-year ambient survey, Indo-Gangetic Plain, India (biomass burning emissions)	Bacteria and fungi	Concentrations of viable fungi were elevated (annual average ∼116 ± 51 CFU m^3^)
Camacho et al. (2018)	Urban and forest fires, Madeira Island, Portugal	Fungal spores	After fire occurrence, air concentrations of fungal spores increased (peaking ∼10 days post-fire)
Kobziar et al. (2018)	Wildland fires (prescribed burns), longleaf and slash pine woodlands, Florida, USA	Bacteria and fungi	Smoke from wildland fire emitted large numbers of culturable fungi and bacteria; ∼80% of the microbial cells in smoke were estimated to be viable and pathogenic fungal taxa were among those aerosolized
Mirskaya & Agranovski (2020)	Biomass combustion (experimental and field)	Bacteria and fungi	Biomass combustion produced elevated concentrations of viable bacterial and fungal aerosols above background; survival of microbes in combustion products was observed and aerosolization mechanisms identified
Moore et al. (2020)	Wildland fires (prescribed burns), longleaf and slash pine woodlands, Florida, USA	Bacteria	Smoke samples had ∼5x higher cell concentrations (∼80% viable) than ambient air and correlated with particulate matter; biological INPs (ice nucleating particles) enhanced in smoke.
Laws et al. (2021)	Wildfire management operations in Central Valley of California	Fungi, *Coccidioides*	Cluster of coccidioidomycosis cases in wildland firefighter crew; dust/soil disturbance tasks associated with illness, suggesting risk of viable pathogen exposure during wildland fire work
Kobziar et al. (2022)	Wildland fires (prescribed burn), sub-alpine fir forests, Central Rockies, USA	Bacteria and fungi	Smoke samples had ∼4 × higher cell concentrations than ambient air; ∼78% of microbes in smoke inferred to be viable. Viable microbes confirmed
Bonfantine et al. (2024)	Wildland fires (prescribed burn), sub-alpine fir forests, Central Rockies, USA	Bacteria	Source–sink modeling: 70% of bacterial taxa in smoke traced to local vegetation/soil; smoke-borne microbes contribute ∼25% to terrestrial sinks *vs* <4% from ambient air
Ellington et al. (2024)	Grassland fires and lab burns, tallgrass prairie, Kansas, USA	Bacteria and fungi	Smoke-mediated dispersal of viable microbes to soils; Viable microbes confirmed

### Smoke-borne pathogen ecology in the broader context of disease ecology

To understand the implications of smoke-borne bioaerosols, we position smoke-borne pathogen ecology in the broader field of disease ecology. This review synthesizes evidence about how wind and fire interact to influence pathogen dispersal, highlighting key similarities with and differences from other forms of airborne pathogen transport.

**Figure 3:**
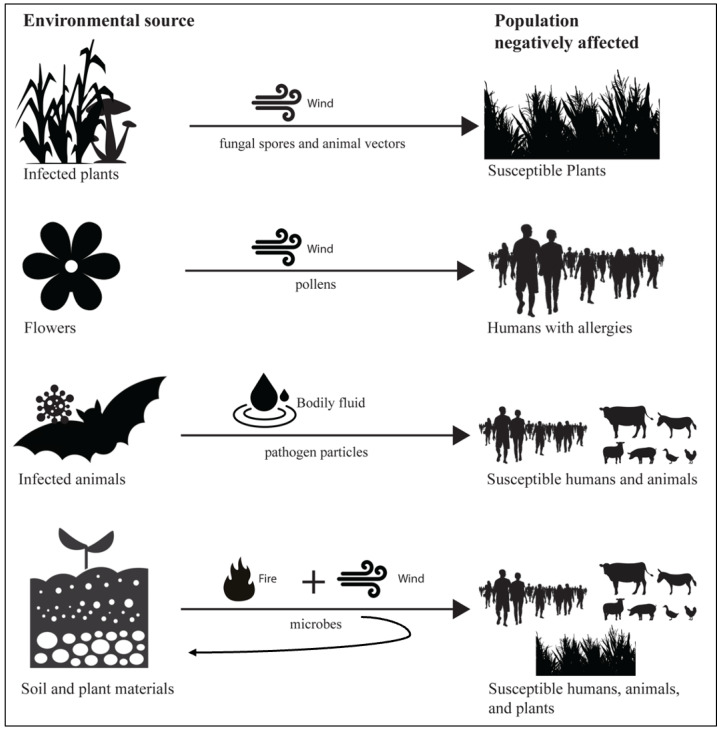
Smoke-borne pathogen ecology accounts for a unique combination of components in disease ecology. There are many examples of wind dispersal of pathogens from one host individual to another closely related host individual. Wind dispersal of pollen links plants to human health *via* allergies to pollen. Zoonotic disease links wild and domestic animal populations to human health. However, smoke-borne pathogens have the unusual feature of being released from soil and plant materials with the potential to infect humans, animals, and plants. Enhanced aerosolization of both surface and endosphere microbes from plants and soil surfaces caused by fires, coupled with strong vertical transport processes, has the potential to disperse pathogens across great distances to hosts, and the potential for deposition in new environmental reservoirs.

Smoke-borne pathogen ecology has unique features in disease ecology, in terms of the combination of the dual role of wind and fire and the unusual dispersal of pathogens from soil and plant materials, potentially to a broad range of hosts, spanning humans and animals, as well as plants (Fig. 3). Wind is an important factor in the epidemiology of many human and animal pathogens (Ellwanger & Chies, 2018). Wind dispersal is common for plant pathogens, such as *Puccinia melanocephala* (causing sugarcane rust) and *P. graminis* (causing wheat stem rust), potentially carrying fungal spores between continents (Brown & Hovmøller, 2002). Plant-to-human effects are exemplified by exposure of allergic humans to wind-borne pollen (allergens), and are relevant to common respiratory allergic diseases, such as hay fever and asthma (Ravindra, Goyal & Mor, 2022). While many infectious diseases z (*e.g.*, rabies from animals, salmonellosis from poultry) have well established routes of cross-species transmission from animals to human, pathogens such as *Histoplasma* and *Coccidioides* illustrate how environmental fungi in soil may be aerosolized *via* fire with the potential to infect humans (Haselow et al., 2014; Phillips et al., 2023). These examples illustrate the conceptual link between smoke-borne pathogens and host infection, and the novel features of smoke-borne pathogen ecology.

Smoke-borne pathogen ecology combines uplift driven by fire combustion and wind dispersal, which creates the unique aerosolization process, including potential pathogen spread from plants, litter, and soils to humans and other hosts. There is the potential for a number of factors to interact to influence mobilization of microbial species: incomplete combustion, convective wind-driven aerosolization, and vertical lofting. Likewise, many factors such as temperature, combustion efficiency, and atmospheric conditions may interact to determine which microbes survive lofting and transport. There is the potential for smoke-borne pathogens to be transported long distances (Mims & Mims, 2004) with smoke, but systematic data to trace pathogens through source to sink transport, as well as data about post-deposition viability and infection potential, were not available. Ellington et al. (2024) used laboratory methods to show that bacteria can be moved from fuels into soils, where they colonize and begin metabolic functioning and competing with native species. Genetic source-tracking methods can be used to gain insight into the relationship between the fuels and soils in burn and deposition zones and the composition of smoke (*e.g.*, Bonfantine et al., 2024), but do not reveal whether organisms survive the translocation. New studies are needed to broadly assess microbial and pathogen viability post-dispersal as a foundation for assessing the potential for infection of distant hosts.

### The role of fire in pathogen uplift and dispersal

Wildland fires alter microbial communities at both local and landscape scales, with fire behavior, vegetation structure, and atmospheric conditions influencing the composition of microbial assemblages that are aerosolized in smoke plumes (Kobziar et al., 2024b). Similarly, wildland fire severity and frequency are determined by factors such as vegetation structure, composition, and phenological stage, climate and weather, and topography (Curt, Borgniet & Bouillon, 2013). Wildland fires result from the convergence of ignitions, fuel availability, and suitable weather conditions, with the duration, extent, and severity of fires being influenced by the complex factors that drive fire behavior and effects (Pausas & Paula, 2012; Pausas & Keeley, 2021; Kobziar et al., 2024a). These physical fire processes differentially affect the aerosolization of terrestrial microbes from soil and vegetation sources within and even nearby a fire perimeter (Kobziar et al., 2024b; Bonfantine et al., 2024; Ellington et al., 2024). Smoke-borne microbial assemblages are closely linked with the vegetation and soil microbiomes at the fire site, as shown by Bonfantine et al. (2024) who found that 70% of smoke-borne bacteria came from soils and fuels in the burn site, based on 16S rRNA sequencing. It is hypothesized that the taxonomic composition of aerosolized microbial assemblages also varies according to fire behavior factors like fire intensity, consumption rate, and combustion efficiency—along with fuel type, weather, and other environmental factors (Kobziar et al., 2022; Kobziar et al., 2024a; Kobziar et al., 2024b). Some existing research has addressed this hypothesis, showing that composition and abundance or concentrations of DNA-containing bacteria differed depending on the fuel types burned in laboratory experiments (Moore et al., 2020; Kobziar et al., 2018). Laboratory burns characterized by smoldering *versus* flaming combustion also differed in their production of microbes (Kobziar et al., 2018). Higher-intensity or larger fires can generate significant convection columns, increasing the probability of long-range transport of smoke and its constituents (Pausas & Keeley, 2021). However, the role of fire behavior and smoke dynamics in pathogen dispersal is underexplored, particularly regarding how fire and smoke conditions shape microbial viability and endurance beyond the initial emission processes.

Once aloft, the survival and transport of microbes is governed by atmospheric parameters specific to that altitude, including windspeed, humidity, temperature, and the presence or absence of nutrients and stressors (Haddrell & Thomas, 2017). Factors such as the potential for cloud formation and wet deposition also drive microbial transport and deposition. While efforts have been made to model and predict smoke-borne bacterial transport (Kobziar et al., 2024b) and seasonal air-connectivity patterns that may explain plant pathogen aerial dispersal (Choufany et al., 2021), explicit modeling of the movement of fungal spores in wildland fire smoke remains unexplored, including the role of spore traits. Given the diversity of fungal spore characteristics, and challenges in quantifying concentrations of spores of specific taxa, their transport in the atmosphere is difficult to predict. In addition, aerosolized microbes are likely impacted by atmospheric processes; for example, microbial cells may act as nuclei for water condensation in clouds during dispersal (Morris et al., 2013; Moore et al., 2020; Kobziar et al., 2022), and basidiospores recruit water droplets in the air and can promote rainfall (Hassett, Fischer & Money, 2015). These mechanisms may influence deposition and survival processes, along with gravitational settling and impaction processes (Fröhlich-Nowoisky et al., 2012).

The potential for microbes in smoke plumes to travel vast distances depends on fire behavior (which drives the degree of initial uplift either beyond or below the mixing layer) and subsequent atmospheric conditions, with the potential for transport across thousands of kilometers. For example, particulate matter measured in Texas was associated with fungal spore concentrations, inferred to have come from smoke plumes from wildland fires 1,500 km away in the Yucatán Peninsula (Mims & Mims, 2004). Smoke plumes can cause substantial atmospheric changes, including updraft winds capable of lifting spores and microbes. For example, during the El Portal fire in Yosemite National Park, updraft winds reached speeds of 13.5 m/s (Lareau & Clements, 2017), which may have enhanced the release of spores, hyphae, and bacterial cells from both fuels and soils.

Fuel type, composition, and loading help to determine the nature of wildland fires and the subsequent distribution of smoke. In forests with high tree density and vertical connectivity, fires often escalate to crown fires, which are high-intensity events affecting all vegetation layers (Keeley & Syphard, 2020). In contrast, surface fires in grasslands and savannas spread through the herbaceous layer, and are usually lower intensity but can cover large areas (Pausas & Paula, 2012). Organic matter resulting from the accumulation of dead vegetation in peatlands is prone to smoldering fires that can persist underground, releasing smoke over extended periods (Keeley & Syphard, 2020). Crown fires and megafires are often driven by high fuel loads and extreme weather conditions, and produce large and tall plumes, such as pyrocumulonimbus (PyroCb) clouds, that may carry particles over long distances (Pausas & Keeley, 2021). The Australian “Black Summer” wildland fires of 2019–2020 generated unprecedented pyrocumulonimbus activity, injecting significant amounts of aerosols into the stratosphere, which were observed to persist and circulate around Antarctica (Tencé et al., 2022). These pyrocumulonimbus plumes also exhibited unique characteristics such as rapid ascent to altitudes above 31 km, anticyclonic circulations, and unusual chemical compositions (Kablick III et al., 2020). These intense fires create strong convection columns. In contrast, surface fires and smoldering fires generate lower energy, but at times longer-duration smoke episodes, which may result in more localized dispersal of smoke particles including microbes (Kobziar et al., 2018). Each of these types of fire is likely to produce different microbial assemblages and result in different microbial assemblages being transported across long distances, and with corresponding different probabilities of transporting living pathogens depending on the chemical and physical profile of the smoke. Bioaerosol research has shown that a wide range of factors determine whether and which microbes (pathogens or others) survive long-distance transport in the air, including the interaction between species traits and the dynamic atmospheric environment (Schmale III & Ross, 2015). Further research is needed to quantify how combustion conditions influence the viability of transported microbes, including pathogens, particularly in high-intensity wildland fires.

### Smoke-borne pathogen concentrations and composition

Published concentrations of microbes emitted in smoke are limited, but consistently show concentrations higher than that of background air. For example, in high-intensity crown fires emissions ranged from 10^4^–10^5^ bacterial cells per cubic meter of air, and in lower-intensity grass and pine litter fires, from 10^3^–10^4^ (Kobziar et al., 2022; Moore et al., 2020). Consistent with this pattern, culturable bacteria during field biomass burns reached ∼2.85  ×  10^3^ CFU m^−^^3^ (*vs.* 1.33  ×  10^3^ CFU m^−^^3^ background), and ash aerosolization yielded ∼3.24  ×  10^3^ CFU m^−^^3^ (*vs.* 650 CFU m^−^^3^ ambient) (Mirskaya & Agranovski, 2020). In prescribed fire smoke air mixtures, total microbial cells averaged 6.7 ± 1.3 × 10^4^ cells m^−^^3^ about fivefold above pre-ignition air with ∼80% inferred viable and culturable taxa detected (Moore et al., 2020).

Only one study we identified enumerated the release of microbes from a given area of terrestrial source burned, Kobziar et al. (2024b). The authors provided estimates of emission at approximately 3.71 × 10^1^^4^ microbial cells per hectare of sub-alpine fir forest burned (Kobziar et al., 2024b). The same study also derived emission factor estimates, which are commonly used in smoke modeling to correlate mass of fuel burned with emissions of gases and particulates from wildland fires (*e.g.*, Urbanski, 2014). Estimates of bacterial emission factors from prescribed burns of both piles of logging slash and forests ranged from 1.39  × 10^10^ to 7.68  × 10^1^^1^ cells per megagram of biomass burned, depending on fuel and fire type, indicating a potentially significant contribution of microbes to wildland fire smoke composition (Kobziar et al., 2024b). By comparison, in ambient air samples collected in Toronto, Canada, fungal spores and pollen made up 12–22% of the organic carbon fraction, and 4–11% of the total particulate mass under 2.5 µm (Womiloju et al., 2003).

The World Health Organization recently released a fungal priority pathogens list (WHO-FPPL) emphasizing the need to increase surveillance, bridge knowledge gaps, and strengthen public health interventions for 19 human fungal pathogens (World Health Organization, 2022). In this list, two of the critical fungal pathogens, *Cryptococcus neoformans* and *Aspergillus fumigatus*, can be aerosolized and infect humans *via* inhalation, with the potential to spread through wildland fire smoke.

**Figure 4:**
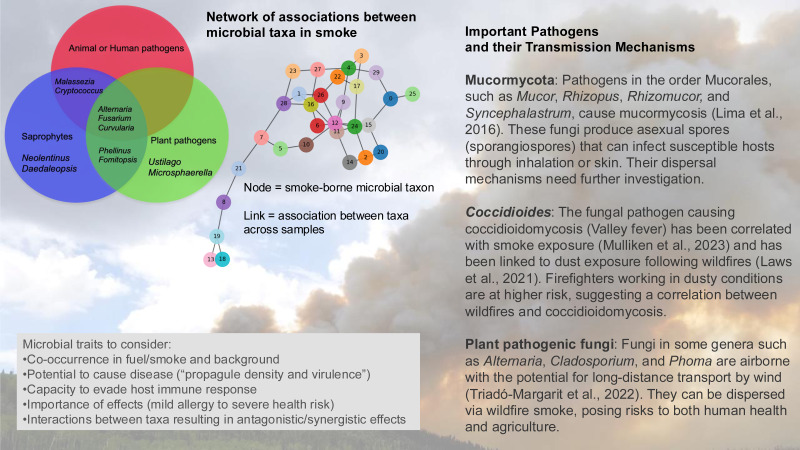
The microbial assemblage in smoke from wildland fires or controlled burns includes potential pathogens of humans, animals, and plants. Analysis of the network of associations between taxa may identify indicator species for the presence or absence of pathogens, and potentially provide information about associations in the source plant and soil material. OTU, operational taxonomic unit. (Background photo credit: L. Kobziar).

There is the potential to identify important smoke-borne pathogens based on understanding how microbial traits—such as heat tolerance, UV resistance, and dormancy mechanisms—influence survival and long-range dispersal (Fig. 4). Some pathogenic propagules such as spores are resistant to UV radiation and desiccation and can remain viable during long-distance dispersal (Wyatt, Wösten & Dijksterhuis, 2013). Dry spore-discharging fungi can be emitted with surface winds as low as 1 m/s (Elbert et al., 2007) and having smaller spores can facilitate wind dispersal of fungal taxa over longer distances and time periods.

Fire-tolerant fungal traits include resistance to heat, the ability to persist as dormant propagules, and the rapid colonization of post-fire environments, which can help certain fungi thrive in burned areas. Fox et al. (2022) classified fungi into groups based on their response to fire, in terms of traits such as fire responsiveness (the ability to capitalize on the environmental changes brought about by fire) and fire resistance. Fire can impact mycorrhizal fungi, reducing overall richness, while some taxa like *Pustularia* may become dominant after high-severity fires (Pulido-Chavez et al., 2021). Similarly, fungal pathogens such as *Teratosphaeria* spp. can become more prevalent in burned plots in pine savanna ecosystems (Semenova-Nelsen et al., 2019), where there are opportunities to infect woody plant hosts that have fire injuries. Pathogenic fungi that produce survival spores or other structures are examples of fire resistance (Fox et al., 2022). Among bacteria, *Bacillus* spp. form heat-resistant endospores (Baron, 1996), and *Staphylococcus aureus can* enter a dormant state with reduced metabolic activity under harsh conditions (such as fire) and resume growth when favorable conditions return (Nwabor, Leejae & Voravuthikunchai, 2021). Both of these genera were found in wildland fire smoke in existing pyroaerobiology studies (Kobziar et al., 2022; Bonfantine et al., 2024; Ellington et al., 2024). However, the degree to which smoke-altered microbial communities contribute to pathogen virulence and disease outbreaks remains a research gap.

## Discussion

### Research gaps and prospects

A One Health perspective is important for understanding smoke-borne pathogen ecology, addressing the complex interactions among communities of pathogens and other microbes, wildland fire dynamics, and potential hosts. The dispersal of smoke-borne pathogens has the potential to be influenced by combustion processes, while sources may affect the composition, concentration, viability and infectious potential of aerosolized microbial assemblages. Microbiological methods such as culturing can assess microbe survival post-aerosolization, determining post-fire viability (Yao & Mainelis, 2007). Prescribed burns may not represent all fuel types and fire behaviors, but they can provide a controlled environment to study and model these effects under moderate weather conditions, and the application of transcriptomic analyses during such experiments could reveal microbial functional traits such as stress tolerance, virulence, and metabolic activity that determine survival and dispersal in smoke. In 2018, prescribed fire burns were implemented on an area (6.8 million acres) comparable to wildland fires (8.7 million acres) in the United States (National Interagency Coordination Center, 2019). As an example, the Fire and Smoke Model Evaluation Experiment (FASMEE; Prichard et al., 2019) in the Fishlake National Forest of Utah, has enabled sampling of smoke microbe emissions and transport during large (>900 acres) high-intensity crown fires (*e.g.*, Aurell et al., 2021; Bonfantine et al., 2024; Kobziar et al., 2022; Kobziar et al., 2024b). Still, these unique burns are not precisely analogous to large wildland fires which can burn many thousands of acres under extreme weather conditions in a single day. To further our understanding of smoke-borne pathogen transport, a wide net must be cast to enable study, ideally during prescribed burns, wildfires, and agricultural burning.

**Figure 5:**
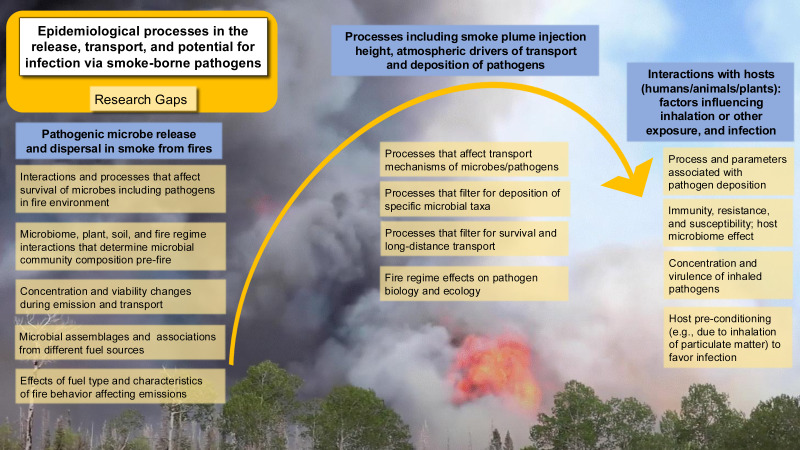
Key epidemiological processes and corresponding research gaps in smoke-borne pathogen ecology. Pathogens are released from soil and plants, dispersed in smoke plumes, deposited across landscapes, and may infect human, animal, or plant hosts. Research gaps are highlighted at each step of the process to guide future studies and risk mitigation strategies. (Background photo credit: L. Kobziar).

To build a framework for the ecology and epidemiology of smoke-borne pathogens, we identify seven priority areas to address the critical research areas and knowledge gaps as discussed below, each of which presents open questions and opportunities for further investigation (Fig. 5). The integration of a One Health approach is particularly important here. Understanding smoke-borne pathogens requires evidence from aerobiology, fire ecology, atmospheric science, microbiology, plant pathology, veterinary medicine, and public health. Without cross-disciplinary synthesis, we risk underestimating how smoke-borne microbes contribute to disease burdens across human, animal, and plant systems.

Note that this scoping study is limited to published journal articles and available grey literature, without including unpublished data or ongoing research and surveillance efforts. We did not perform formal risk-of-bias assessment, and our keyword searches may have missed studies outside the databases evaluated. Few studies directly report smoke-borne microbial infection and pathogenic outcomes, so many of our identified research needs remain speculative, albeit grounded in analogous evidence from aerobiology and disease ecology.

### Microbial interactions with smoke-borne pathogens

There is limited information about how microbial communities interact in smoke plumes and whether these interactions influence pathogen viability and dispersal dynamics. Open questions include:

• Do pathogen species and individual cells frequently co-occur on the same smoke particles, or do they tend to remain separate throughout transport?
• What pre-fire interactions in soil and plant materials influence which microbial species become aerosolized?
• Do some microbial traits promote co-dispersal, increasing the likelihood of cross-kingdom interactions?
• Do some associations during deposition change the risks of infection?

Air sampling of wildfire smoke poses significant methodological challenges. Concentrations of viable microbes in wildland fire smoke are inherently hard to reach and often in very low concentrations (similar to all bioaerosols), making airborne microbial sampling difficult (Kobziar et al., 2018). Patterns of co-occurrence of pathogens on individual smoke particles is a research gap. Aerosolized microbes may undergo selection pressures during emission, transport, or both; this may potentially favor spore-forming and stress-tolerant taxa, although such trends in smoke-borne microbial composition have not yet been reported (Kobziar et al., 2022). Investigating how microbial associations shift during dispersal and deposition could help predict infection risks and ecological consequences. Evaluating microbiome interaction networks can indicate how pathogens co-occur in smoke plumes, possibly showing the fingerprint of biological interactions in plant and soil materials before fires. The study of microbiome networks can identify associations that might reflect synergistic or antagonistic relationships between microbes and system traits (Poudel et al., 2016; Poudel et al., 2023), potentially informing predictions about pathogen viability and dispersal and identifying useful indicator taxa for pathogens.

### Infection rates of smoke-borne pathogens

Current wildland fire smoke surveillance frameworks focus almost exclusively on abiotic pollutants (*e.g.*, PM_2.5_, ozone), without epidemiological screening for microbial content. Existing air quality monitoring networks, such as EPA’s AirNow or the interagency IMPROVE and CSN networks (Federal Land Manager Environmental Database, 2024) provide critical data on particulate exposure but do not assess biological hazards. Understanding infection potential is fundamental to determining whether smoke-borne microbes pose a significant health threat to humans, animals, and plants. While viable microbes have been detected in wildland fire smoke, their ability to cause infection in hosts has not been reported. Key questions include:

• Is infectivity affected by fire’s aerosolization or smoke’s transport processes?
• Are viable smoke-borne pathogen concentrations high enough to cause infection?
• How does the pathogen concentration threshold differ across infectious agents and host conditions (*i.e.,* immunocompromised *vs.* competent individuals)
• Does smoke exposure damage host tissues in ways that facilitate (or reduce) infection (Reid et al., 2016)?

Physical and chemical injuries from smoke inhalation may increase susceptibility to systemic infections or, conversely, may trigger immune responses that reduce infection rates. The synergistic effects of smoke exposure and microbial infections remain poorly characterized, and further research such as genetic network analysis and gene set enrichment analysis (Fang et al., 2013) may help to determine whether smoke-borne pathogens exacerbate respiratory or systemic diseases.

### Host immunity and susceptibility

The relationship between host immunity and smoke-borne infections remains largely unexplored. Wildland fire smoke contains a complex mixture of particulate matter, toxins, and microbes, all of which can influence host immune responses. Critical research gaps include:

• Are certain animal or plant species more vulnerable to smoke-borne pathogens?
• How do behavioral responses to wildland fire smoke (*e.g.*, avoidance, exposure duration) influence infection risks?
• Does inhalation of smoke-borne microbes alter host immune functions in ways that increase or decrease disease susceptibility?

Several pathogens, such as *Pseudomonas syringae* pv. tomato and *Alternaria infectoria*, can bypass both animal and plant immune systems and cause disease in hosts across kingdoms (Kim et al., 2020). Further studies are needed to understand how these cross-kingdom pathogens spread (Van Baarlen et al., 2007) and determine how smoke exposure influences host immune system function and whether exposure increases susceptibility to novel or opportunistic infections. Population genetics studies can identify host genetic variation that influences susceptibility to smoke-borne pathogens.

### Interactions with host microbiome

Microbial communities residing on plant surfaces, in animal respiratory tracts, and in soil ecosystems play a critical role in regulating pathogen colonization and infection outcomes (Du, Han & Tsuda, 2025). However, the impact of smoke-borne microbes on host microbiomes remains an open research question. Specific areas for investigation include:

• How do smoke-borne pathogens interact with the pre-existing microbiome of hosts, especially the human lung microbiome?
• How do smoke-borne plant pathogens interact with plant surface microbiomes on deposition, affecting disease progress?
• Do smoke-transported pathogens displace or interact with beneficial microbes, leading to secondary health effects?

Recent research suggests that disruptions to the host microbiome could influence infection outcomes and disease severity (Natalini, Singh & Segal, 2023). Analyzing the network of associations between smoke-borne pathogens and the host’s existing microbiome can shed light on potential disruptions or synergistic effects that may influence disease outcomes. Studies addressing these questions could inform and generate strategies to mitigate infection risks.

### Impact on different human populations

Wildland fire smoke exposure is not uniform across human populations, and certain groups are disproportionately affected due to geographic, occupational, and socioeconomic factors (Black et al., 2017) which may translate into differential smoke-borne pathogen exposure and health risks. Key questions include:

• How do the traits of human populations (*e.g.*, race, gender, ethnicity, socioeconomic status and comorbidities) influence health risks due to wildland fire smoke and smoke-borne pathogens?
• Are certain occupational groups (*e.g.*, firefighters, agricultural workers) at higher risk of smoke-borne pathogen exposure?
• Does access to protective measures (*e.g.*, N-95 masks, air filtration, housing quality) affect susceptibility to smoke-associated diseases?

People working in high-exposure jobs may have greater vulnerability to infections. For example, firefighters are routinely exposed to wildland fire smoke, and outbreaks of Coccidioidomycosis (Valley fever) among wildland firefighters suggest a potential occupational health hazard (Laws et al., 2021). If smoke-borne pathogens are confirmed to influence health risks, studies should assess whether these pathogens contribute to increased disease burdens in high-risk populations.

### Influence of wildland fire environment on pathogen assemblages

The composition of smoke-borne microbial assemblages is shaped by multiple source and fire-related variables, yet there is limited knowledge about how different fire conditions influence microbial dispersal patterns. Open questions include:

• How do the many dynamic variables associated with the wildland fire environment affect the composition, concentration, and viability of smoke-borne microbial and pathogen assemblages?
• How do meteorological factors (temperature, humidity, wind speed) influence pathogen transport distances?
• Do specific wildland fire conditions favor certain pathogen groups, increasing their prevalence in post-fire deposition environments?

Fungal germination rates can be reduced by smoke exposure in some species (Parmeter Jr & Uhrenholdt, 1976). However, the broader implications for pathogen survival, deposition patterns, and reintroduction into ecosystems remain unknown. Addressing these gaps could help develop predictive models for pathogen spread *via* wildland fire smoke.

### Preventing smoke-borne infections

Potential mitigation strategies for smoke-associated infections are currently underdeveloped, largely due to gaps in knowledge regarding transmission pathways and risk factors. Interventions at various stages, including pathogen reproduction, release, dispersal, and infection processes, could reduce infection risk. If the potential role of these pathogens is confirmed, evaluating the feasibility and impact of these interventions will be important. To build a management framework, informing strategies to mitigate disease risk, research would be needed to evaluate the feasibility of

• **Targeted fuel reduction strategies in high-risk agricultural or wildland fire areas to minimize pathogen aerosolization**: Identifying locations with a high risk of releasing pathogens that can infect vulnerable crop, wild plant, human, or animal hosts would be an important step. Prioritization could help allocate resources effectively. Prescribed burns have been shown to alter microbial communities (Kobziar et al., 2024a). Although direct measurements of smoke-borne microbes under prescribed burn regimes are scarce, evidence from particulate matter studies suggests that prescribed burns may lead to lower but more persistent smoke concentrations in nearby areas compared to wildfires (Navarro et al., 2018), which implies a longer duration of exposure potentially relevant for microbial dispersal dynamics.
• **Pathogen monitoring in wildland fire smoke**: Once the role of smoke-borne pathogens is better understood, implementing monitoring systems to detect pathogens in wildland fire smoke could provide early warnings and risk assessments for at-risk populations.

## Conclusions

This scoping review highlights smoke-borne pathogen ecology in the broader context of wildland fire dynamics, disease ecology, and the One Health framework. By synthesizing current research, we discussed some mechanisms of microbial aerosolization, transport, and deposition in wildland fire smoke, identified key pathogen traits that facilitate survival and dispersal, and outlined critical research gaps that must be addressed to assess potential health and ecological risks. The interdisciplinary nature of smoke-borne pathogen research underscores the importance of integrating fire ecology, aerobiology, microbiology, and epidemiology in a One Health framework to fully understand the implications of wildland fire-driven pathogen dispersal.

While existing pyroaerobiology studies provide evidence for transport of viable microbes in smoke, fundamental questions remain regarding pathogen infection potential, interactions with host immune systems and microbiomes, and the role of wildland fire smoke in disease outbreaks across human, animal, and plant populations. We identified seven priority research areas needed to bring together the current fragmented evidence across disciplines in a One Health framework. Beyond identifying gaps, our review emphasizes actionable priorities including integrating microbial surveillance into existing wildfire smoke monitoring networks, improving and standardizing air-sampling methods to assess pathogen viability, and developing predictive models that combine fire behavior, atmospheric dynamics, and microbial ecology to guide risk assessments. If new evidence for health risks from smoke-borne pathogens becomes available, other responses should focus on advancing detection methods, monitoring pathogen movement in wildland fire smoke, and evaluating the effectiveness of interventions such as fuel management strategies and public health protective measures.

The practical implications of this scoping study are clear: public health agencies, agricultural biosecurity programs, and wildfire management agencies should begin considering microbes as part of wildland fire risk assessments. Updated fire management strategies, such as occupational health protocols like the recently-announced N-95 mask protocol for US federal firefighters, can help reduce exposure and enhance preparedness. Incorporating a One Health approach, researchers can develop comprehensive frameworks that integrate human, animal, and environmental health considerations, ensuring a full understanding of wildland fire-driven microbial dispersal and its consequences.

##  Supplemental Information

10.7717/peerj.20605/supp-1 Supplemental Information 1 PRISMA Checklist

## Additional Information and Declarations

### Data Availability

The following information was supplied regarding data availability: This is a literature review.
